# Nucleofection optimization and *in vitro* anti-tumourigenic effect of TRAIL-expressing human adipose-derived mesenchymal stromal cells

**DOI:** 10.1186/s12935-014-0122-8

**Published:** 2014-11-26

**Authors:** Kamal Shaik Fakiruddin, Puteri Baharuddin, Moon Nian Lim, Noor Atiqah Fakharuzi, Nurul Ain Nasim M Yusof, Zubaidah Zakaria

**Affiliations:** Stem Cell Laboratory, Haematology Unit, Cancer Research Centre, Institute for Medical Research (IMR), 50588 Kuala Lumpur, Malaysia

**Keywords:** Human adipose derived mesenchymal stromal cells, Nucleofection, TNF-related apoptosis inducing ligand (TRAIL), Cancer cell lines, Proliferation, Apoptosis

## Abstract

**Background:**

Tumour homing capacity of engineered human adipose-derived mesenchymal stromal cells (ADMSCs) expressing anti-tumour agents might be the key for a much safer and yet efficient targeted tumour therapy. However, ADMSCs exhibit resistant to most gene transfection techniques and the use of highly efficient viral vectors has several disadvantages primarily concerning safety risk. Here, we optimized the use of highly efficient and safe nucleofection-based transfection using plasmid encoded for TNF-Related Apoptosis Inducing Ligand (TRAIL) into ADMSCs and investigated the potential anti-tumourigenic of TRAIL-expressing ADMSCs (ADMSCs-TRAIL) on selected cancer models *in vitro*.

**Methods:**

Different concentration of TRAIL-encoded plasmid and ADMSCs were nucleofected and the percentage of fluorescence cells were analyzed to determine the optimal condition. TRAIL protein and mRNA were validated in nucloeofected ADMSCs using ELISA and RT-PCR respectively. Evaluation of TRAIL specific death receptors were performed on both tumours (A549/lung tumour, LN18/glioblastoma and HepG2/hepatocellular carcinoma) and haematological malignant lines (REH/acute lymphocytic leukaemia, K562/chronic myelogenous leukaemia and KMS-28BM/multiple myeloma) using flow cytometry. ADMSCs-TRAIL was subsequently assessed for anti-tumourigenic properties using both proliferation assay (MTS assay) and apoptosis assay (Annexin-V / Propidium Iodide staining).

**Results:**

Nucleofection showed increased total plasmid concentration (2 μg to 8 μg) resulted in significantly higher reporter expression (11.33% to 39.7%) with slight reduction on cells viability (~10%). ADMSCs-TRAIL significantly inhibited ~50% of cell proliferation in LN18, signifying sensitivity of the cell to ADMSCs-TRAIL mediated inhibition. Inhibition of both tumour and malignant lines proliferation by ADMSCs-TRAIL conditioned medium noticed in HepG2, A549 and REH respectively, whereas K562 and KMS-28BM malignant lines exhibit resistant to ADMSCs-TRAIL mediated inhibition. Moreover, we found that native ADMSCs alone were capable of inducing apoptosis in both LN18 and HepG2 tumour lines, despite substantial increased on the percentage of apoptosis by ADMSCs-TRAIL.

**Conclusion:**

ADMSCs-TRAIL selectively inhibit cancer model and markedly induces apoptosis. Through investigation of the specific TRAIL death receptors expression, we saw that the receptors expression did influence the sensitivity of some but not all cancer lines to TRAIL-mediated inhibition. This study provides further insight into the anti-tumourigenic potential of ADMSCs-TRAIL on different cancer models.

## Background

Human adipose-derived mesenchymal stromal cells (ADMSCs) are characterized as subset of cells that are multipotent, capable of self-renewal, propagation and differentiation into three mesenchyme lineages (osteogenesis, adipogenesis and chondrogenesis) *in vitro* [1]. Compared to other type of stromal cells, human ADMSCs serve as an attractive candidate for regenerative therapy as these cells are easily isolated, expanded and differentiated to other type of mature cells under specific growth induction. The differentiation ability of ADMSCs has been made not only to three mesenchyme lineages, but also into other type of cells such as hepatocyte [2-4], neuron [5-7] and insulin producing cells [7-10].

Homing characteristic of mesenchymal stromal cells (MSCs) to the tumour microenvironment, suggested that these cells might play a role during tumour development [11-13]. Several studies that performed genetic alteration of ADMSCs found that exogenous expression of therapeutic genes enhanced the treatment efficacy of ADMSCs on the target site [14-17]. Ideally, by utilizing both engineered ADMSCs and tumour homing characteristic of these cells, an efficient site-specific tumour treatment can be generated [18]. Like most primary cell lines, ADMSCs exhibit resistant to classical gene transfection technique and currently the most efficient gene delivery system is through viral transduction. However, the use of virally transduced cells for clinical application has been hampered due to its potential mutation, immune response and ‘safeness’ [19].

The nucleofection technology is a safe non-viral electroporation-based transfection system that combines both specific electrical parameters and cell type transfection solutions to drive plasmid DNA, oligonucleotides and siRNA directly to the cytoplasm and nucleus of the cell [20]. Using this method, high transfection efficiency can be achieved in hard to transfect cells [21]. This method has been successful in primary cells such as neurons [22] and keratinocytes [23]. Despite most studies that presented the method on primary cells, there are few reports on the optimization technique specific on ADMSCs. Thus we studied on the technique based on plasmid concentration, cell number and the exogenous gene expression.

To study the process of nucleofection optimization and also the anti-tumourigenic effects of genetically engineered ADMSCs expressing TRAIL (ADMSCs-TRAIL) on both haematological malignancies and solid tumours, we used the tumour necrosis factor (TNF)-related apoptosis inducing ligand (TRAIL) gene. It is a type II membrane-bound (MB) protein that can be processed by cysteine protease to generate soluble ligand. Both MB protein and soluble ligand can rapidly induce apoptosis in variety of cancers [24]. Although several studies had shown the anti-tumour effect of exogenous TRAIL, systemic bioavailability and the toxicity of the agent hindered the used of this ligand for future clinical application [25]. Nonetheless, by utilizing ADMSCs as a vehicle for stable TRAIL secretion directly to the tumour microenvironment, efficacies of TRAIL therapy can be enhanced and off target toxicity evoke by non-specific TRAIL receptor binding may be overcome.

In the present study, we performed optimization of nucleofection (Amaxa™) based transfection to drive DNA plasmid encoded for TRAIL into ADMSCs (ADMSCs-TRAIL). Two DNA plasmid, pCMV6 (Origene Rockville, MD) and pLOC (Thermo Scientific, Waltham, MA) encoded for TRAIL was compared for reporter expression in HEK293T cells and plasmid with the highest reporter expression was selected for our subsequent analysis. We report for the first time selective inhibition of ADMSCs-TRAIL based on specific cancer type of both haematological and non-haematological malignancies. The objective of this study is to provide further understanding on the potential application of ADMSCs-TRAIL based on different cancer models.

## Results

### Morphological characterization of ADMSCs *in vitro*

**Figure 1:**
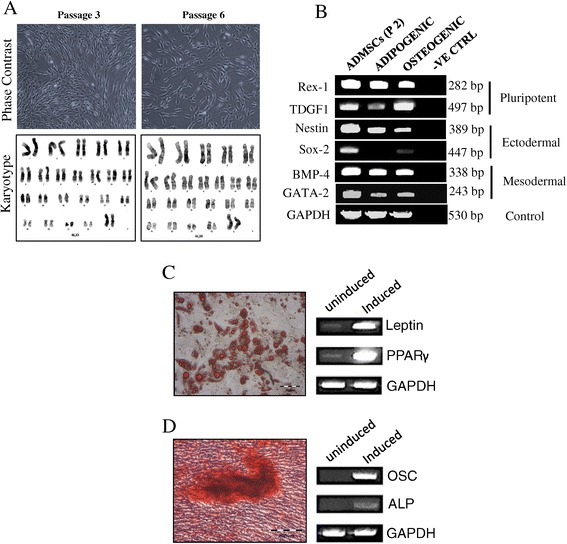
**Characterization of ADMSCs.** Phase contrast of *in vitro* expanded ADMSCs at passage 3 and passage 6 showing both consistent with fibroblast-like morphology (magnification: 10X) **(A)**. Genomic stability was also observed in ADMSCs at passage 3 and passage 6 **(A)**. Semi quantitative RT-PCR analysis of germ specific markers in uninduced ADMSCs (P2) when compared to induced (adipogenic and osteogenic) ADMSCs; template = 5ul, PCR cycle =30, internal control is GAPDH **(B)**. Mesenchymal lineages differentiation of adipogenesis **(C)** stained with Oil Red O and osteogenesis **(D)** stained with Alizarin Red were performed and results of RT-PCR for the relevant transcripts after differentiation were also depicted (left lane: uninduced cells and right lane: induced cells).

The ADMSCs at passage 2 were purchased from the American Type Culture Collection (ATCC, Manassas, USA), grown and expanded using defined ATCC media specific for human mesenchymal stromal cells. The morphology of these cells appeared to be fibroblastic, elongated and spindle shape (Figure 1A). Apart from slight reduction in the proliferation rate, the cells could be expanded up to passage 6, with no apparent morphological changes (Figure 1A). For this study, cells below or at passage 6 were used for all of the experiment. Karyotypic analysis performed at passage 3 and passage 6 showing cells are genomically stable (Figure 1A).

### Specific germ layer markers expression

Semi quantitative reverse transcriptase polymerase chain reaction (RT-PCR) was performed for pluripotent (Rex-1 and TDGF), ectodermal (Nestin and Sox-2) and mesodermal (Bmp-4 and Gata-2) markers expression between differentiated (adipogenic and osteogenic) to non-differentiated ADMSCs. Results were presented with higher pluripotent, ectodermal and mesodermal markers in non-differentiated ADMSCs compared to differentiated cells (Figure 1B).

### Lineages differentiation of ADMSCs

The ADMSCs were induced into adipogenic and osteogenic using specific differentiation medium purchased from ATCC. Adipogenic differentiation of ADMSCs was observed after 21 days cultured in differentiation medium, displayed by intracellular lipid accumulation and verified by Oil Red O staining (Figure 1C). The expression of adipogenic specific transcripts such as leptin and PPARγ was also positive as compared to uninduced cells (Figure 1C). The osteogenic differentiation potential of ADMSCs was confirmed by the calcium deposit characteristic of bone formation detected by Alizarin Red staining (Figure 1D). In addition, the induced cells expressed osteogenic specific transcripts such as osteocalcin (OSC) and alkaline phosphatase (ALP) compared to uninduced cells (Figure 1D).

### Selection for an optimal expression vector

**Figure 2:**
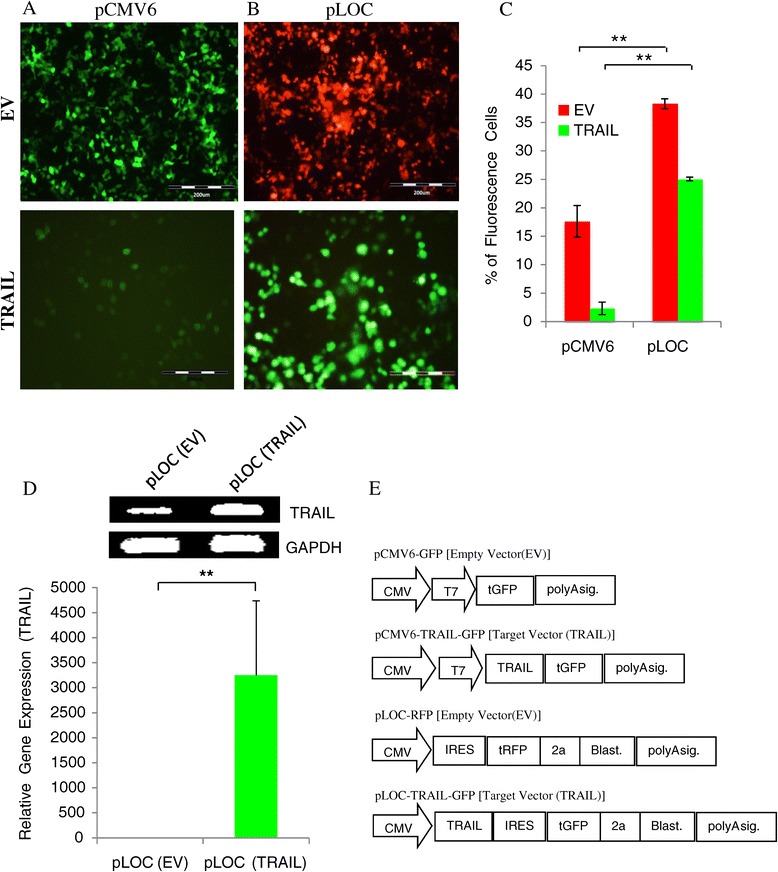
**Selection for the optimal TRAIL expression vectors in HEK293T cells.** HEK293T cells were transiently co-transfected with either pCMV6 **(A)** or pLOC **(B)** plasmid using Fugene 6 (3:1 transfection reagent to DNA ratio) and images obtained by fluorescence inverted microscopy (magnification: 10X). Significantly higher percentage of fluorescence cells after pLOC plasmid transfection compared to pCMV6 for both the empty vector (EV) and TRAIL encoded plasmid as quantified by FACS, **(C)**. Expression of TRAIL transcript was confirmed by quantitative RT-PCR and GAPDH was used as internal control for the analysis **(D)**. Plasmid map for the relevant vectors were also depicted (**E**; **p < 0.001; t-test).

Both plasmid vectors, pCMV6 (Origene) and pLOC (Thermo Scientific) either encoded for TRAIL [target vector (TRAIL)] or vector alone [empty vector (EV)] were transiently co-transfected into HEK293T cells using Fugene6 (Roche) transfection reagents with 3:1 transfection reagent to plasmid ratio, and incubated for 48 hours. Pictures taken from fluorescence optical microscopy revealed empty vector (EV) with high reporter expression for both pCMV6 and pLOC plasmid, however, target vector (TRAIL) presented with lower reporter expression in pCMV6 compared to the pLOC plasmid (Figure 2A-B). Fluorescence quantification on the EV presented with significantly higher (p < 0.001) fluorescence events in pLOC plasmid compared to pCMV6 with 38.29% and 17.64% respectively (Figure 2C). Similarly for the target vector (TRAIL), pLOC plasmid presented with higher fluorescence events (p < 0.001) with 25.09%, compared to pCMV6 plasmid with only 2.34% (Figure 2C). Subsequent quantitative RT-PCR analysis validated the increased of TRAIL gene expression on HEK293T cells after pLOC plasmid transfection (Figure 2D). Based on comparison of reporter expression between both vectors, pLOC plasmid was chosen for our subsequent analysis.

### Increased plasmid concentration enhanced reporter expression in post-nucleofected ADMSCs

**Figure 3:**
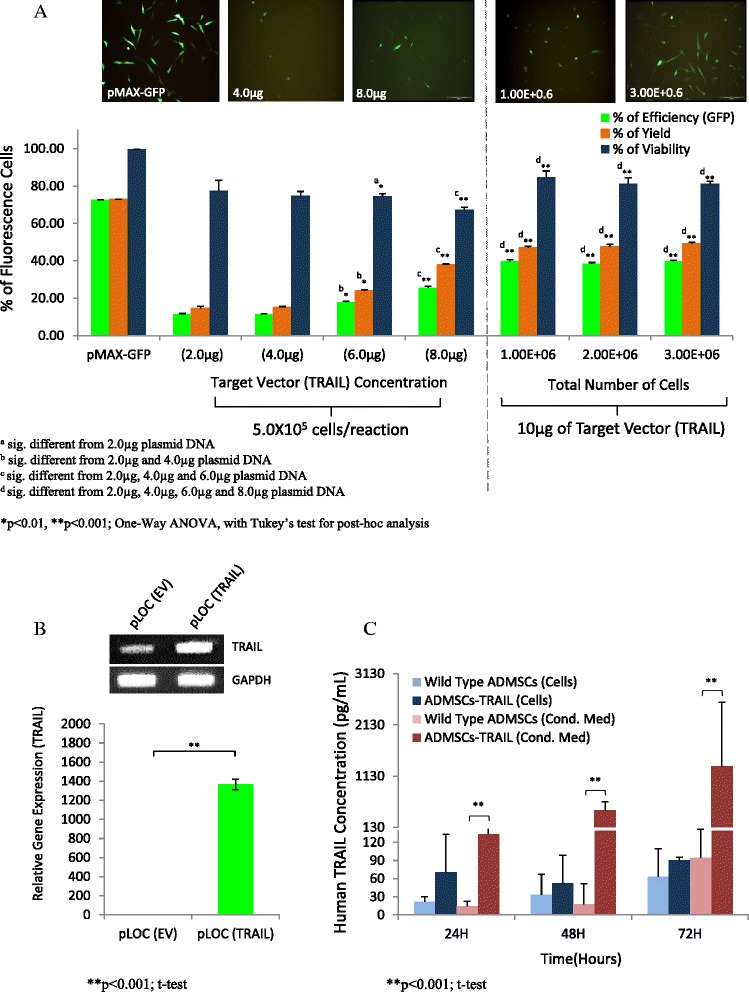
**Nucleofection optimization and validation of TRAIL gene and protein expression in ADMSCs.** Optimization was performed based on different concentration of TRAIL encoded pLOC plasmid and cell number. Higher amount of the plasmid significantly increased percentage of efficiency and yield, with reduction on cells viability **(A)**. However, increased total number of cells with the highest plasmid concentration enhanced cellular viability of the nucelofected ADMSCs **(A)**. A substantially high level of TRAIL mRNA **(B)** and secreted protein expression **(C)** validated exogenous TRAIL expression in nucleofected ADMSCs as analyzed by quantitative RT-PCR and ELISA respectively .

To further enhanced reporter expression in nucleofection-based transfection, we increased the plasmid concentration of the target vector (TRAIL). Initially, we used manufacturer (Amaxa) recommended protocol consisting of 2 μg plasmid and 5.0 x 10^5^ total cells in a single reaction. However, due to the low reporter expression as quantified by FACS, we increased the plasmid concentration from 2 μg up to 8 μg in a single reaction while maintaining the same number of cells per reaction, and 2 μg of pMAX-GFP plasmid was used as control. As expected, increased plasmid concentration of 8 μg, we managed to achieve higher percentage of events (p < 0.001) resulting in up to 25.49% efficiency and 37.82% yield compared to 11.70% efficiency and 14.67% yield using manufacturer recommended protocol (Figure 3A).

### Increased cell number reduced toxic effects caused by high plasmid concentration

We noticed that even though reporter expression enhanced as plasmid concentration increased, percentage of viability reduced to 67.21% when cells were transfected with the highest plasmid concentration (8 μg per reaction) compared to 87.50% viability when 2 μg of plasmid concentration was used as recommended by the manufacturer (Figure 3A). Thus, we next tested whether percentage of viability, yield and efficiency can be enhanced with higher number of cells. Hence, 10 μg of pLOC plasmid encoded for TRAIL with increased number of cells for up to 3.0 x 10^6^ were used for each reaction. Surprisingly, when nucleofection was performed with the highest number of cells (3.0 x 10^6^ cells per reaction), we observed the percentage of viability enhanced to 81.16%, with percentage of efficiency and yield increased to 39.80% and 49.00% respectively which is ~10% higher compared to the previous analysis (Figure 3A).

### TRAIL-expressing ADMSCs validated by mRNA and protein analysis

Validation of TRAIL gene and protein expression in post-nucleofected ADMSCs was performed using quantitative RT-PCR and ELISA respectively. Substantial increased (p < 0.001) in fold change for TRAIL gene expression between ADMSCs nucleofected with plasmid encoded for TRAIL compared to the empty vector was noticed (Figure 3B). Significantly high (p < 0.001) TRAIL protein was detected between 48 hours to 72 hours in conditioned medium of ADMSCs after pLOC TRAIL nucleofection confirming that the TRAIL protein retained as a secreted ligand between indicated time point for in vitro analysis (Figure 3C).

### High percentage of the TRAIL ligand needed to exert anti-proliferative effects in selected haematological malignant line

**Figure 4:**
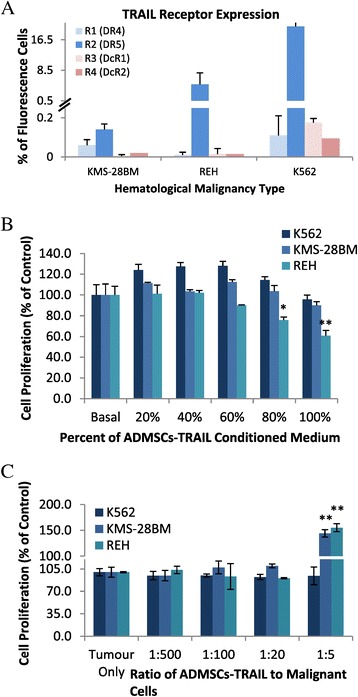
**The anti-proliferative effects of ADMSCs-TRAIL on haematological malignancies as analyzed by MTS assay.** FACS analysis of TRAIL receptors revealed that only R2 (DR5) expression was high in both K562 and REH lines, whereas lower expression of all four receptors was seen in KMS-28BM **(A)**. ADMSCs-TRAIL and its conditioned medium (80% and 100% ) induced significant inhibition of REH proliferation, whereas both K562 and KMS-28BM remained resistant to TRAIL-mediated inhibition **(B)**. Increased ratio of ADMSCs-TRAIL and malignant lines induced significant cellular proliferation noticed in REH and KMS-28BM (**C**; *p < 0.01, **p < 0.001; t-test).

Inhibition of cell proliferation from 100% (basal) to 76% and 61% was noticed in REH line cultured with 80% and 100% ADMSCs-TRAIL conditioned medium (Figure 4B). This result is consistent with the high R2 (DR5) TRAIL receptor expression seen in REH line (Figure 4A). However, high concentration of ADMSCs-TRAIL conditioned medium is needed to induce meaningful effects. In contrast KMS-28BM and K562 lines showed resistance to TRAIL ligand when cells were cultured with ADMSCs-TRAIL conditioned medium (Figure 4B).

### ADMSCs-TRAIL enhanced specific haematological malignant growth

In contrast to the inhibitory effects induced by ADMSC-TRAIL conditioned medium on REH malignant line, direct co-culture with ADMSCs-TRAIL did not induce significant inhibition as shown by Figure 4C. Furthermore, direct co-culture of both REH and KMS-28BM malignant lines with increased number of ADMSCs-TRAIL significantly enhanced their proliferation. K562 showed no changes on its proliferation as the cell were cultured with increased number of ADMSCs-TRAIL.

### Specific tumour lines inhibition by ADMSCs-TRAIL

**Figure 5:**
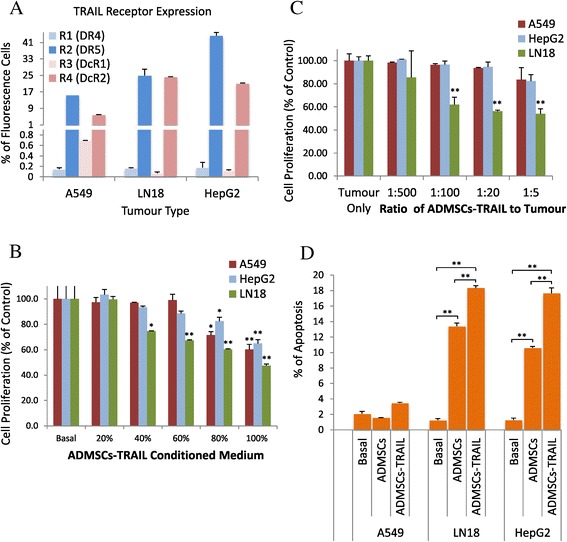
**ADMSCs-TRAIL inhibited proliferation and induced tumour cell apoptosis.** High R2 (DR5) and R4 (DcR2) TRAIL receptors expression was noticed in A549, LN18 and HepG2 lines **(A)**. Proliferation of LN18 was persistently inhibited as the tumour line was cultured with 40% to 100% ADMSCs-TRAIL conditioned medium. However, the inhibition of A549 and HepG2 tumour lines was only noticed when the cells were cultured with 80% and 100% ADMSCs-TRAIL conditioned medium **(B)**. Constant inhibition of LN18 tumour lines noticed when cultured with increased number of ADMSCs-TRAIL **(C)**. Native ADMSCs alone were able to induce apoptosis in both LN18 and HepG2; in addition, the apoptotic effect was enhanced when the tumour cells were cultured with ADMSCs-TRAIL (**D**; *p < 0.01, **p < 0.001; t-test).

We further evaluated the anti-proliferative effect of ADMSCs-TRAIL and its conditioned medium on selected panel of solid tumour lines. Our result indicates consistent tumour inhibition from 100% cell proliferation to 75%, 67% and 48%, noticed in LN18 cultured with 40%, 60% and 100% ADMSCs-TRAIL conditioned medium respectively (Figure 5B). Persistent inhibition of LN18 proliferation was also noticed when the tumour cell cultured with increased ratio of ADMSCs-TRAIL as shown by Figure 5C, suggesting LN18 is highly sensitive to TRAIL-mediated inhibition. High percentage of conditioned medium (80% - 100%) was needed to exert anti-proliferative effects in both A549 and HepG2 as shown by Figure 5B with no apparent changes on tumour cells proliferation when cultured with increased number of ADMSCs-TRAIL (Figure 5C).

### TRAIL secretion by ADMSCs enhanced tumour apoptosis induced by native ADMSCs

Direct co-culture using transwell assay was performed to further validate the induction of apoptosis by TRAIL secreting ADMSCs. Induction of apoptosis was noticed on both LN18 and HepG2 cultured with the native ADMSCs with 13.41% and 10.60% respectively (Figure 5D). Moreover, the apoptotic effect was significantly enhanced to 18.32% and 17.65% for both LN18 and HepG2 respectively when these cells were cultured with ADMSCs-TRAIL (Figure 5D). The percentage of apoptosis was slightly increased when A549 line was cultured with the ADMSCs-TRAIL as compared to native ADMSCs (Figure 5D).

## Discussion

The tumour homing capacities of MSCs have led to the profound idea of developing genetically engineered ADMSCs expressing anti-tumour agents as a cell based vector system. This on site specific targeted therapy may later be applied in patients to enhance treatment efficacy and reduce systemic toxicity [26]. However, to produced genetically engineered ADMSCs expressing death ligand is a challenge, and like most primary cells, ADMSCs remain resistance to genetic alteration. In this study, we report optimization of the nucleofection-based transfection using TRAIL gene on ADMSCs and evaluated the anti-tumourigenic effects of TRAIL-expressing ADMSCs on both haematological and non haematological malignant lines. ADMSCs were characterized and validated based on cellular morphology, genomic stability, germ layers mRNA expression and mesenchymal lineages differentiation (Figure 1). pLOC was selected over pCMV6 plasmid vector for subsequent transfection analysis on ADMSCs based on the high level of reporter expression observed in HEK293T (Figure 2). We believe that the IRES (internal ribosome entry site) sequence, which is part of the pLOC plasmid DNA, might enhance transgene expression in HEK293T cells as shown by Figure 2E [27]. We performed optimization of nucleofection-based transfection using different concentration of TRAIL encoded pLOC plasmid with increased ADMSCs cell number per reaction. Higher plasmid concentration of 8 μg increased the reporter expression up to 2 fold with moderate reduction on cell viability (Figure 3A). However, we manage to substantially enhanced cell viability when total number of cells was increased to 3.0 x 10^6^ cells per reaction and this directly improved efficiency of the reporter expression and yield (Figure 3A). This observation suggested that higher number of cells may reduce toxicity effect induced by the plasmid. In addition, we also verified the mRNA and protein expression of TRAIL in nucleofected ADMSCs using quantitative RT-PCR and ELISA respectively (Figure 3B-C).

Binding of the TRAIL ligand to the intracellular region of R1 (DR4) and R2 (DR5) receptors leads to the recruitment of death-inducing signalling complex (DISC) and the activation of the death signal by apoptosis [28]. In contrast, high expression of R3 (DcR1) and R4 (DcR4) will compete for the binding of the TRAIL ligand to the R1 and R2 receptors, resulted in blocking of TRAIL-mediated apoptosis and leading to insensitivity of the TRAIL therapy to the tumour [28]. Study on the anti-tumourigenic effects of secreted TRAIL ligand by ADMSCs-TRAIL on haematological malignancy correlated to the R2 (DR5) receptor expression as shown by KMS-28BM and REH lines. Lowest R2 (DR5) receptor expression in KMS-24BM may have caused the cells resistant to TRAIL-mediated inhibition (Figure 4B). In contrast, high expression of the R2 (DR5) receptor expression in REH caused the cells sensitive to TRAIL-mediated inhibition when the cells were cultured with the highest percentage of ADMSCs-TRAIL conditioned medium (Figure 4B). However, the presence of R2 (DR5) may not necessarily correlate to the sensitivity of the malignant cells to TRAIL as shown in K562 lines (Figure 4A), indicating some cells may have high expression of apoptotic resistance genes or low expression of caspases mediated cell death that circumvent TRAIL-mediated apoptosis [29]. High expression of the R3 (DcR1) and R4 (DcR2) seen in K562 cell line compared to other haematological malignant lines may also contribute to the resistance phenotype of this cells to TRAIL-mediated inhibition (Figure 4A).

MSCs are known for its ability as immune modulator capable of regulating proliferation and differentiation of immune cells such as macrophages, B and T cells, NK cells, and even mast cells [30]. Studies suggested that MSCs promote cellular proliferation and maintenance of either normal or malignant hematopoietic cells in direct co-culture [31,32]. Our results indicated TRAIL secretion by ADMSCs induced cellular inhibition of hematopoietic malignant cell noticed in REH line shown by Figure 4B. However, direct cell to cell contact between ADMSCs-TRAIL and leukemic lines namely REH and KMS-28BM may circumvent the inhibitory effects induced by secreted TRAIL due to the strong properties of native MSCs to promote hematopoietic malignant survival. Therefore, we believed precautions should be taken if ADMSCs expressing anti-tumour ligand is to be applied in the context of haematological malignancies.

Consistent tumour inhibition and apoptosis seen in LN18 cultured with ADMSCs-TRAIL and its conditioned medium signifying that the cell is highly sensitive to TRAIL-mediated inhibition (Figure 5B-D). Furthermore, as indicated in Figure 5D, significantly high induction of apoptosis by native ADMSCs co-cultured directly with both LN18 and HepG2 was observed, while TRAIL-secreting ADMSCs substantially induced higher percentage of apoptosis on both lines. These results might be due to the secreted factors (cytokines and chemokines) by MSCs that influence the anti-tumourigenic effects in several cancer models [33]. Correlated to the high R2 (DR5) receptor expression and also the high percentage of apoptosis noticed in HepG2 tumour line, this cell may appear to be highly sensitivity to ADMSCs-TRAIL mediated inhibition. However, there is no apparent change on HepG2 tumour cells proliferation when these cells were cultured with ADMSCs-TRAIL (Figure 5C). This result suggested that ADMSCs-TRAIL does induced cytotoxicity by binding of the TRAIL ligand secreted by ADMSCs-TRAIL to the R2 (DR5) receptor on HepG2 as analyzed by the apoptosis assay (Figure 5D). However it may not necessarily induced cytostaticity to the proliferation of HepG2 cells, as the cells may still be highly proliferative and resistance to TRAIL-mediated inhibition. HepG2 tumour line has been known for its resistance phenotype to TRAIL-mediated inhibition as reported by several papers [34-36]. In fact, there are also few papers that shown apoptosis may not certainly effect cellular proliferation as these two processes are independent to each other [37,38]. High expression of the R2 (DR4) receptor as seen in A549 theoretically indicated that this cells are highly sensitive to TRAIL-mediated apoptosis (Figure 5A). However, our results showed that the anti-proliferation effect of TRAIL ligand is only exerted as the cells were cultured with high concentration of ADMSCs-TRAIL conditioned medium (Figure 5B). Moreover, slight reduction of A549 proliferation was noticed when the cell was cultured with increased number of ADMSCs-TRAIL with no apparent proliferative inhibition (Figure 5C). We believed that this is due to the competitive binding of the TRAIL ligand to R1 and R2 receptors by high expression of both R3 (DcR1) and R4 (DcR2) receptor that reduced TRAIL-mediated apoptosis in A549 shown by Figure 5A.

In summary, our data demonstrated that nucleofection is efficient to deliver TRAIL gene into ADMSCs. However, suitable plasmid design, optimal plasmid concentration and cell number are needed to achieve an ideal condition for the technique. We believe that the R2 (DR5) receptor expression does not inevitably signify the sensitivity of the malignant cells to TRAIL-mediated apoptosis as observed in K562, A549 and HepG2 cell lines model. Moreover, the properties of native ADMSCs do play an essential role in the induction of anti-tumourigenic effect and TRAIL secretion by ADMSCs-TRAIL may enhance ADMSCs-mediated tumour inhibition as noticed in both LN18 and HepG2 lines. In the framework of haematological malignancies, due to the contradicting effect of ADMSCs-TRAIL and its conditioned medium, we believed therapeutic exploitation of ADMSCs-TRAIL in the context of haematological malignancies would not be suitable as it may enhance the survival of haematological malignant cells. Finally, we concluded that more functional assessments are still needed if anti-tumour targeted strategy utilizing ADMSCs-TRAIL is to be developed in the near future.

## Materials and methods

The research protocol was approved by our institutional review boards (Medical Research Ethics Committee/MREC, Ministry of Health Malaysia).

### Cell lines

The human adipose-derived mesenchymal stromal cells (ADMSCs) at passage 2 were purchased from the American Type Culture Collection (ATCC, Manassas, USA) and HEK293T was courtesy of Dr Alan Khoo Soo Beng (Molecular Pathology Unit, Institute for Medical Research, Malaysia). Solid tumour lines [A549 (lung tumour), LN18 (glioblastoma) and HepG2 (hepatocellular carcinoma)] were purchased from ATCC. Non-adherent hematopoietic malignant lines [REH (acute lymphocytic leukaemia), K562 (chronic myelogenous leukaemia)] and KMS-28BM (multiple myeloma) were purchased from ATCC and JCRB cell bank respectively.

### Cell culture

The ADMSCs were cultured in specific MSCs growth medium purchased from ATCC, supplemented with 2% fetal bovine serum (FBS), 5 ng/mL recombinant basic fibroblast growth factor (rh FGF), 5 ng/mL rh FGF acidic, 5 ng/mL recombinant epidermal growth factor (rh EGF), 10 μg/mL gentamicin, 0.25 μg/mL amphotericin B, 10 Units/mL penicillin and 33 μM phenol red, (all purchased from ATCC, Manassas, USA). Eight percent of fetal bovine serum (ATCC) was added later into the complete medium to make 10 serum concentration as complete working medium. The cells were incubated in a humidified incubator at 37°C supplied with 5% carbon dioxide, routinely maintained in 75 cm^2^ tissue culture flasks and harvested when cells reached 80-90% confluence using 0.05% trypsin-EDTA (ATCC) for subculturing and expansion.

The HEK293T cells were cultured in DMEM high glucose with sodium pyruvate supplemented with 10% FBS and 100 IU/ml penicillin, 100 μg/ml streptomycin (all from Invitrogen Corporation, Carlsbad, CA). The cultures were maintained in 5% CO_2_ in a humidified incubator at 37°C. When the cells reached 80%–90% confluence, the cells were harvested with 0.25% trypsin-EDTA (Invitrogen Corporation) and subcultured for expansion.

Cancer lines (A549, LN18, HepG2, REH, K562 and KMS-28BM) were cultured in RPMI-1640 supplemented with 10% FBS and 100 IU/ml penicillin, 100 μg/ml streptomycin, 1% non essential amino acids (NEAA) and 1% L-Glutamine (all from Invitrogen Corporation, Carlsbad, CA). The cultures were maintained in 5% CO_2_ in a humidified incubator at 37°C. When adherent tumour cells reached 80%–90% confluence, cells were harvested with 0.25% trypsin-EDTA (Invitrogen Corporation) and subcultured for expansion.

### Karyotyping analysis

Genomic stability of ADMSCs in passage 3 and passage 6 was investigated using the conventional karyotyping, and analyzed using the standard G-banding technique. Metaphase spreads were prepared using standard procedure of 10 μg/ml colcemide treatment for 4 hours, followed by 0.075 M potassium chloride hypotonic treatment and methanol/glacial acetic acid fixation (3:1) ratio. GTG-banding using TrypLE™ Express (Gibco by Life Technologies, Grand Island, NY, USA) pre-treatment was performed according to established protocol. Cytogenetic analysis was performed using Cytovision 7.3 software. Microscopy was performed using optical bright field, Olympus BX 50. Ten G-banded metaphases were analyzed for each sample.

### Reverse transcription polymerase chain reaction (RT– PCR) analysis

Expression of pluripotent (Rex-1 and TDGF) and germ layer specific (BMP-4, GATA-2, Nestin and SOX-2) markers were evaluated between non-differentiated ADMSCs at passage 2 and differentiated (adipogenic and osteogenic) cells. To validate differentiation of ADMSCs into specific lineages, expression of genes related to adipogenic (Leptin and PPARγ) and osteogenic (osteocalcin and ALP) was performed. Total RNA was extracted using RNAeasy kit (Qiagen Hamburg GmbH, Germany) according to the manufacturer’s protocol. The extracted RNA was quantified at 260 nm absorbance, and purity of RNA was evaluated from the 260/280-absorbance ratio using the spectrophotometer (BioPhotometer Plus, Eppendorf, Hamburg, Germany). First strand cDNA was synthesized using the Transcriptor First Strand cDNA synthesis kit (Roche Applied Science, Nonnenwald, Penzberg, Germany) according to manufacturer protocol. PCR were performed with primers (Table 1) and PCR kit (Promega, Madison, WI, USA) on a thermocycler (Eppendoff Mastercycler gradient, Hamburg, Germany). Initial denaturation was performed at 94°C for 5 min, followed by denaturation at 94°C for 30 s, annealing at 55°C for 30 s, and extension at 72°C for 1 min for 30 cycles. A final extension of 10 min at 72°C was also performed for each reaction. The PCR products were analyzed on 1.2% FlashGel™ agarose electrophoresis system (Cambrex, USA). GAPDH was used as an internal control.

**Table 1:** **Human primer sequences used for RT-PCR**

**Gene**	**Accession**	**Sense primer**	**Antisense primer**	**Product Size (bp)**
Nestin	NM_006617.1	CAGCGTTGGAACAGAGGTTGG	TGGCACAGGTGTCTCAAGGGTAG	389
BMP 4	NM_130851.2	GTCCTGCTAGGAGGCGCGAG	GTTCTCCAGATGTTCTTCG	338
Rex-1	NM_174900	GCGTACGCAAATTAAAGTCCAGA	ATCCTAAACCAGCTCGCAGAAT	282
Gata-2	NM_001145661.1	AGCCGGCACCTGTTGTGCAA	TGACTTCTCCTGCATGCACT	243
TDGF	NM_003212.3	GCCCGCTTCTCTTACAGTGTGATT	AGTACGTGCAGACGGTGGTAGTTCT	497
Sox 2	NM_003106.3	CCCCCGGCGGCAATAGCA	TCGGCGCCGGGGAGATACAT	447
Leptin	NM_000230.2	TGTGCCCATCCAAAAAGTCC	GTTTGGAGGAGACTGACTGCG	100
PPARγ	NM_015869.4	GAGCACTTCACAAGAAATTACC	AATGCTGGAGAAATCAACTG	151
ALP	NM_000478.4	GGAAGGGTCAGTCAGGTT	GTGGGCCGCTCTAGGCACCAA	500
Osteocalcin	NM_199173.4	ATGAGAGCCCTCACACTCCTC	GCCGTAGAAGCGCCGATAGGC	294
TRAIL	NM_003810.2	TTGTTGATGAAAAGTGCTAGAAATA	ATGGTCCATGTCTATCAAGTGC	150
GAPDH	NM_002046.4	TGAAGGTCGGAGTCAACGGATT	CATGTGGGCCATGAGGTCCACCAC	530

### Quantitative reverse transcription polymerase chain reaction (qRT– PCR)

Quantitative gene expression of TRAIL was evaluated in both HEK293T and ADMSCs after plasmid transfection and nucleofection respectively. Initially, total RNA was extracted and evaluated for purity as previously described. First strand cDNA was synthesized using the Transcriptor First Strand cDNA synthesis kit (Roche Applied Science, Nonnenwald, Penzberg, Germany) according to manufacturer protocol. The qRT-PCR reaction was prepared using SYBR 1 master mix (Roche Applied Science, Nonnenwald, Penzberg, Germany) and primers as stated (Table 1). Quantitative RT-PCR was performed using the lightcycler 480 (Roche) under the following cycle conditions; Pre-denaturation for 4 mins at 95°C followed by 40 cycles consisting of denaturation at 95°C for 15 sec, annealing at 60°C for 30 sec and extension at 72°C for 30 sec followed by dissociation curve. The basic relative gene expression (RQ) was calculated manually (refer below) and the efficiency (E) of primer binding equal to 2.

$$ \mathbf{Relative}\ \mathbf{expression}\ \left(\mathbf{R}\mathbf{Q}\right) = \frac{{{\mathrm{E}}_{\mathrm{Target}}}^{\Delta \mathrm{C}\mathrm{p}\ \mathrm{Target}\ \left(\mathrm{Mean}\ \mathrm{C}\mathrm{p}\ \mathrm{C}\mathrm{ontrol}\ \hbox{--}\ \mathrm{Mean}\ \mathrm{C}\mathrm{p}\ \mathrm{Sample}\right)}}{{{\mathrm{E}}_{\mathrm{Reference}}}^{\Delta \mathrm{C}\mathrm{p}\ \mathrm{Reference}\ \left(\mathrm{Mean}\ \mathrm{C}\mathrm{p}\ \mathrm{C}\mathrm{ontrol}\ \hbox{--}\ \mathrm{Mean}\ \mathrm{C}\mathrm{p}\ \mathrm{Sample}\right)}} $$

- Target: Target genes analyzed (TRAIL)
- Reference: Reference Genes (GAPDH)
- Sample: HEK293T and/or ADMSCs (Transfected with pLOC-TRAIL encoded plasmid)
- Control: HEK293T and/or ADMSCs [Transfected with pLOC-EV (Empty Vector) plasmid]

### Adipogenic and osteogenic differentiation

The ADMSCs were induced for adipogenic and osteogenic differentiation using specific lineage differentiation medium (ATCC, Manassas, USA). Passage 3 ADMSCs were seeded at 1.7 × 10^5^ cells/well in 6 wells tissue culture plates (BD) for 24 hours. The next day, medium was replaced with specific adipogenic and osteogenic differentiation medium. Cells were induced for 21 days with medium being changed once every alternate day. After 21 days, the adipogenic and osteogenic cultures were fixed and stained with Oil Red O (0.3%) and Alizarin Red solution respectively. The stained cells were examined under an inverted microscope immediately after staining. Expression of adipogenesis (leptin and PPARγ) and osteogenesis (osteocalcin and ALP) markers was also assessed using RT–PCR.

### Plasmids construct

Plasmid vectors (pCMV6 and pLOC) either with or without the TRAIL coding sequence were purchased from Origene (Rockville, USA) and Thermo Scientific (Waltham, MA) respectively. For the empty vector (EV), both pCMV6 and pLOC were tagged either with green fluorescence protein (GFP) and red fluorescence protein (RFP) respectively, whereas for the TRAIL encoded plasmids, both pLOC and pCMV6 consisted of only GFP tag protein. Both plasmids utilized cytomegalovirus (CMV) immediate early promoter to drive transgene expression. pCMV6 plasmid vector consist of C-terminal for mammalian protein expression, while pLOC plasmid consist of the IRES (internal ribosome entry site) sequence for simultaneous expression of both transgene and fluorescence tag proteins.

### HEK293T transfection

Transfection using the lipid-based system namely Fugene6 (Roche) in HEK293T using both pCMV6 and pLOC plasmid was performed following procedures recommended by manufacturer. In brief, cells were seeded in six-well plates at a density of 5.0 × 10^5^ cells per well and allowed to grow overnight. Transfection complex, consisting of 2 μg of plasmid and 6 μl of transfection reagents, was directly added to the wells in the presence of serum-containing medium, and cells were assayed 48 hours later for reporter expression using flow cytometry.

### ADMSCs nucleofection

The Nucleofection of ADMSCs was performed according to the manufacture recommendation (Amaxa Biosystem, Cologne, Germany) and pMAX-GFP plasmid was used as control. Briefly, different cell number and plasmid concentration of the TRAIL encoded pLOC plasmid were suspended in 100 μl of human mesenchymal nucleofection solution (Amaxa Biosystem), and pulsed with U23 (high efficiency) program. Immediately after nucleofection, cells were transferred into pre-warmed fresh complete medium in six-well plates and left to grow overnight. The next day, medium was discarded and cells were washed few times to remove debris. Cells were left to grow for another 48 hours before medium was harvested and stored at −80°C for further downstream application. Cells were subjected to fluorescence activated cell sorting (FACS) 72 hours post-nucleofection.

### Analysis of transfection efficiency, viability and yield

Analysis of fluorescence cells in both HEK293T and ADMSCs-TRAIL was performed using flow cytometry. Briefly, cells were detached from 6 wells plate by 5 minute 0.25% trypsin-EDTA incubation, recovered by centrifugation and washed in PBS containing 2% FBS. FACS Calibur instrument (Becton Dickinson BD) was performed and a total of 10,000 events were acquired for data analysis by using Cell Quest software (BD, San Jose, CA). Nonspecific fluorescence was determined using wild type (non-transfected) cells. Percentage of efficiency (GFP^+^) was calculated based on the total percentage events gated on the FL-1 channel. Viability of cells post transfection was assessed using propidium iodides (PI) staining and evaluation was based on (GFP^+^/PI^−^) and (GFP^−^/PI^−^) events gated on both FL-1 and FL-2. Percentage of yield was calculated based on the events gated on (GFP^+^/PI^−^) area and compared to plated number of cells. Fluorescence cells was observed at 20× magnification using CKX31 (Olympus, USA) inverted microscope.

### Enzyme-linked immunosorbent assay (ELISA)

To assess the production of TRAIL secreted protein in ADMSCs-TRAIL, supernatants and cell lysis were collected at different times after nucleofection and assayed in duplicates for TRAIL concentration using ELISA kit (R&D Systems Inc., Minneapolis) according to manufacturer’s instruction.

### Proliferation assay/MTS assay

The MTS assay [3-(4,5-dimethylthiazol-2-yl)-2H-tetrazolium, inner salt] purchased from Promega was used to evaluate the growth inhibitory effects of ADMSCs-TRAIL and its conditioned medium on haematological malignant and solid tumour lines by direct co-culture system. During the execution of the experiments, all of the cells (ADMSCs-TRAIL and cancer lines) were grown in MSCs complete medium for culture standardization. Briefly, 50 μl of cancer lines were seeded in 96 wells plate (Corning Inc, Manassas, VA) at a density of 1.0 × 10^4^ cells/well in 100 μl and left to grown overnight. Either increased number of ADMSCs-TRAIL (20, 100, 500, 2000 total cells/50 μl fresh MSCs medium) based on ratio or 50 μl of different percentage ADMSCs-TRAIL conditioned medium (20%-100%) was added into the wells and incubated for 48 hours. Four hours before the end of the experiment, 20 μl of MTS solution was added into each well and the absorbance at 490 nm was measured using Odyssey® SA Imaging System (Li-Cor, Lincoln, USA), using wells without cells as blank. Proliferation of cancer lines was calculated as follows: Cell Proliferation (% of control) = (OD_490_ of stimulated malignant cells/OD_490_ of unstimulated malignant cells) × 100%.

### Apoptosis assay (annexin v and propidium iodide/PI) staining

Apoptotic induction of ADMSCs-TRAIL on solid tumour lines was performed using the annexin v/pi double staining kit purchased from (Becton Dickinson BD). For the analysis, direct co-cultured between both ADMSCs-TRAIL and tumour cells were performed using 24 wells plate, 0.4 μm transwell system for physical separation of both tumours and ADMSCs-TRAIL (SPL Life Sciences). Briefly, 1.0 × 10^5^ cells/well of ADMSCs-TRAIL and ADMSCs were seeded on the insert and the same number of tumours lines (1.0 × 10^5^ cells/well) were seeded on the bottom wells and co-cultured directly. 48 hours later, tumour cells was harvested by trysinization and collected by centrifugation. Cell palette was suspended in 100 μl of 1× annexin v binding buffer (Becton Dickinson BD) and 5 μl of annexin-v-FITC was added. Antibody incubation was performed at 4°C for 20 minutes and 1 μl of PI was later added before FACS aquisition. Stained cells were subjected to flow cytometric analysis using FACS Calibur instrument (Becton Dickinson BD) and a total of 10,000 events were acquired and analyzed using Cell Quest software (Becton Dickinson BD).

### Statistical analysis

Results are expressed as means ± SD (standard deviation) of three independent experiments. Statistical analysis was performed using the IBM SPSS statistic, version 21. Comparison between two groups was performed using the two-tailed t-test with P values of <0.01 were considered statistically significant. Comparison between groups were performed using one factor analysis of variance (ANOVA) followed by the Tukey’s post hoc.

## Abbreviations

ADMSCs: Adipose-derived mesenchymal stromal cells
TRAIL: Tumour necrosis factor related apoptosis inducing ligand
MSCs: Mesenchymal stromal cells
PPARγ: Peroxisome proliferator-activated receptor gamma
ALP: Alkaline phosphatase
RNA: Ribonucleic Acid
cDNA: Complimentary Deoxyribonucleic acid
qRT-PCR: Quantitative reverse transcriptase polymerase chain reaction
GFP: Green fluorescence protein
RFP: Red fluorescence protein
IRES: Internal ribosome entry site
ELISA: Enzyme link immunosorbent assay
MTT: Methyl-tetrazolium inner salt
PI: Propidium iodide

## References

[1] Barry FP, Murphy JM (2004). Mesenchymal stem cells: clinical applications and biological characterization. Int J Biochem Cell Biol.

[2] Aurich H, Sgodda M, Kaltwaßer P, Vetter M, Weise A, Liehr T, Brulport M, Hengstler JG, Dollinger MM, Fleig WE, Christ B (2009). Hepatocyte differentiation of mesenchymal stem cells from human adipose tissue in vitro promotes hepatic integration in vivo. Gut.

[3] Seo MJ, Suh SY, Bae YC, Jung JS (2005). Differentiation of human adipose stromal cells into hepatic lineage in vitro and in vivo. Biochem Biophys Res Commun.

[4] Banas A, Teratani T, Yamamoto Y, Tokuhara M, Takeshita F, Quinn G, Okochi H, Ochiya T (2007). Adipose tissue-derived mesenchymal stem cells as a source of human hepatocytes. Hepatology.

[5] Ferroni L, Gardin C, Tocco I, Epis R, Casadei A, Vindigni V, Mucci G, Zavan B (2013). Potential for neural differentiation of mesenchymal stem cells. Adv Biochem Eng Biotechnol.

[6] Safford KM, Hicok KC, Safford SD, Halvorsen Y-DC, Wilkison WO, Gimble JM, Rice HE (2002). Neurogenic differentiation of murine and human adipose-derived stromal cells. Biochem Biophys Res Commun.

[7] Gao S, Zhao P, Lin C, Sun Y, Wang Y, Zhou Z, Yang D, Wang X, Xu H, Zhou F, Cao L, Zhou W, Ning K, Chen X, Xu J (2014). Differentiation of human adipose-derived stem cells into neuron-like cells which are compatible with photocurable three-dimensional scaffolds. Tissue Eng A.

[8] Li J, Zhu L, Qu X, Li J, Lin R, Liao L, Wang J, Wang S, Xu Q, Zhao RC (2013). Stepwise differentiation of human adipose-derived mesenchymal stem cells toward definitive endoderm and pancreatic progenitor cells by mimicking pancreatic development in vivo. Stem Cells Dev.

[9] Bhonde RR, Sheshadri P, Sharma S, Kumar A (2014). Making surrogate beta-cells from mesenchymal stromal cells: perspectives and future endeavors. Int J Biochem Cell Biol.

[10] Dave SD, Vanikar AV, Trivedi HL (2014). In-vitro generation of human adipose tissue derived insulin secreting cells: up-regulation of Pax-6, Ipf-1 and Isl-1. Cytotechnology.

[11] D'Souza N, Burns JS, Grisendi G, Candini O, Veronesi E, Piccinno S, Horwitz EM, Paolucci P, Conte P, Dominici M: **MSC and Tumors: Homing, Differentiation, and Secretion Influence Therapeutic Potential**. *Adv Biochem Eng/Biotechno* 2012. 10.1007/10_2012_150 22990585

[12] Stagg J (2008). Mesenchymal stem cells in cancer. Stem Cell Rev.

[13] Sun XY, Nong J, Qin K, Warnock GL, Dai LJ (2011). Mesenchymal stem cell-mediated cancer therapy: A dual-targeted strategy of personalized medicine. World J Stem Cells.

[14] Shahrokhi S, Daneshmandi S, Menaa F (2014). Tumor necrosis factor-alpha/CD40 ligand-engineered mesenchymal stem cells greatly enhanced the antitumor immune response and lifespan in mice. Hum Gene Ther.

[15] Bahmani B, Roudkenar MH, Halabian R, Jahanian-Najafabadi A, Amiri F, Jalili MA: **Lipocalin 2 decreases senescence of bone marrow-derived mesenchymal stem cells under sub-lethal doses of oxidative stress.** *Cell stress & chaperones* 2014. 10.1007/s12192-014-0496-5 PMC4147076 24452457

[16] Hajizadeh-Sikaroodi S, Hosseini A, Fallah A, Estiri H, Noormohammadi Z, Salehi M, Ghaderian SM, Akhavan Niyaki H, Soleimani M, Kazemi B: **Lentiviral Mediating Genetic Engineered Mesenchymal Stem Cell for Releasing IL-27 as a Gene Therapy Approach for Autoimmune Diseases.** *Cell journal* 2013, **16**(3). PMC4204184 24611150

[17] Wang ZH, Li XL, He XJ, Wu BJ, Xu M, Chang HM, Zhang XH, Xing Z, Jing XH, Kong DM, Kou XH, Yang YY (2014). Delivery of the Sox9 gene promotes chondrogenic differentiation of human umbilical cord blood-derived mesenchymal stem cells in an in vitro model. Brazilian journal of medical and biological research = Revista brasileira de pesquisas medicas e biologicas/Sociedade Brasileira de Biofisica [et al.].

[18] Sasportas LS, Kasmieh R, Wakimoto H, Hingtgen S, van de Water JA, Mohapatra G, Figueiredo JL, Martuza RL, Weissleder R, Shah K (2009). Assessment of therapeutic efficacy and fate of engineered human mesenchymal stem cells for cancer therapy. Proc Natl Acad Sci U S A.

[19] Thomas CE, Ehrhardt A, Kay MA (2003). Progress and problems with the use of viral vectors for gene therapy. Nat Rev Genet.

[20] Gresch O, Engel FB, Nesic D, Tran TT, England HM, Hickman ES, Körner I, Gan L, Chen S, Castro-Obregon S, Hammermann R, Wolf J, Müller-Hartmann H, Nix M, Siebenkotten G, Kraus G, Lun K (2004). New non-viral method for gene transfer into primary cells. Methods.

[21] Zaragosi L-E, Billon N, Ailhaud G, Dani C (2007). Nucleofection Is a Valuable Transfection Method for Transient and Stable Transgene Expression in Adipose Tissue-Derived Stem Cells. Stem Cells.

[22] Aluigi M, Fogli M, Curti A, Isidori A, Gruppioni E, Chiodoni C, Colombo MP, Versura P, D'Errico-Grigioni A, Ferri E, Baccarani M, Lemoli RM (2006). Nucleofection is an efficient nonviral transfection technique for human bone marrow-derived mesenchymal stem cells. Stem Cells.

[23] Distler JHW, Jüngel A, Kurowska-Stolarska M, Michel BA, Gay RE, Gay S, Distler O (2005). Nucleofection: a new, highly efficient transfection method for primary human keratinocytes*. Exp Dermatol.

[24] Almasan A, Ashkenazi A (2003). Apo2L/TRAIL: apoptosis signaling, biology, and potential for cancer therapy. Cytokine Growth Factor Rev.

[25] Bellail AC, Qi L, Mulligan P, Chhabra V, Hao C (2009). TRAIL agonists on clinical trials for cancer therapy: the promises and the challenges. Rev Recent Clin Trials.

[26] Nakamura K, Ito Y, Kawano Y, Kurozumi K, Kobune M, Tsuda H, Bizen A, Honmou O, Niitsu Y, Hamada H (2004). Antitumor effect of genetically engineered mesenchymal stem cells in a rat glioma model. Gene Ther.

[27] Mahon MJ (2011). Vectors bicistronically linking a gene of interest to the SV40 large T antigen in combination with the SV40 origin of replication enhance transient protein expression and luciferase reporter activity. BioTechniques.

[28] Sheridan JP, Marsters SA, Pitti RM, Gurney A, Skubatch M, Baldwin D, Ramakrishnan L, Gray CL, Baker K, Wood WI, Goddard AD, Godowski P, Ashkenazi A (1997). Control of TRAIL-induced apoptosis by a family of signaling and decoy receptors. Science.

[29] Hao XS, Hao JH, Liu FT, Newland AC, Jia L (2003). Potential mechanisms of leukemia cell resistance to TRAIL-induced apopotosis. Apoptosis: Int J Programmed Cell Death.

[30] Klopp AH, Gupta A, Spaeth E, Andreeff M, Marini F (2011). Concise review: Dissecting a discrepancy in the literature: do mesenchymal stem cells support or suppress tumor growth?. Stem Cells.

[31] Tabe Y, Jin L, Mills GB, Tsutsumi-Ishii Y, Andreeff M, Konopleva M (2004). Mesenchymal Stem Cells Promote Survival of Leukemic Cells Via Integrin-Linked Kinase (ILK)-Dependent Akt and STAT3 Activation: Implications for Leukemia Therapy. ASH Ann Meet Abstr.

[32] Rodríguez-Pardo VM, Aristizabal JA, Jaimes D, Quijano SM, de los Reyes I, Herrera MV, Solano J, Vernot JP (2013). Mesenchymal stem cells promote leukaemic cells aberrant phenotype from B-cell acute lymphoblastic leukaemia. Hematol/Oncol Stem Cell Ther.

[33] Dai LJ, Moniri MR, Zeng ZR, Zhou JX, Rayat J, Warnock GL (2011). Potential implications of mesenchymal stem cells in cancer therapy. Cancer Lett.

[34] He S-Q, Rehman H, Gong M-G, Zhao Y-Z, Huang Z-Y, Li C-H, Zhang W-G, Chen X-P (2007). Inhibiting survivin expression enhances TRAIL-induced tumoricidal activity in human hepatocellular carcinoma via cell cycle arrest. Cancer Biol Therapy.

[35] Luan Z, He Y, He F, Chen Z: **Rocaglamide overcomes tumor necrosis factorrelated apoptosisinducing ligand resistance in hepatocellular carcinoma cells by attenuating the inhibition of caspase8 through cellular FLICElikeinhibitory protein downregulation**. *Molecular medicine reports* 2014. 10.3892/mmr.2014.2718 PMC4237083 25333816

[36] Kim EY, Yu JS, Yang M, Kim AK (2013). Sub-toxic dose of apigenin sensitizes HepG2 cells to TRAIL through ERK-dependent up-regulation of TRAIL receptor DR5. Mole Cells.

[37] Hecht E, Zago M, Sarill M, Rico de Souza A, Gomez A, Matthews J, Hamid Q, Eidelman DH, Baglole CJ: **Aryl hydrocarbon receptor-dependent regulation of miR-196a expression controls lung fibroblast apoptosis but not proliferation.** *Toxicol Appl Pharmacol* 2014. 10.1016/j.taap.2014.08.023 25178717

[38] Martin KR, Wooden A (2012). Tart cherry juice induces differential dose-dependent effects on apoptosis, but not cellular proliferation, in MCF-7 human breast cancer cells. J Med Food.

